# How to Improve the Early Diagnosis of *Trypanosoma cruzi* Infection: Relationship between Validated Conventional Diagnosis and Quantitative DNA Amplification in Congenitally Infected Children

**DOI:** 10.1371/journal.pntd.0002476

**Published:** 2013-10-17

**Authors:** Jacqueline Bua, Bibiana J. Volta, Alina E. Perrone, Karenina Scollo, Elsa B. Velázquez, Andres M. Ruiz, Ana M. De Rissio, Rita L. Cardoni

**Affiliations:** Instituto Nacional de Parasitología (INP) Dr. M. Fatala Chaben, Paseo Colón 568 (1063), Administración Nacional de Laboratorios e Institutos de Salud (ANLIS) Buenos Aires, Argentina; Institute of Tropical Medicine, Belgium

## Abstract

**Background:**

According to the Chagas congenital transmission guides, the diagnosis of infants, born to *Trypanosoma cruzi* infected mothers, relies on the detection of parasites by INP micromethod, and/or the persistence of *T. cruzi* specific antibody titers at 10–12 months of age.

**Methodology and Principal Findings:**

Parasitemia levels were quantified by PCR in *T. cruzi*-infected children, grouped according to the results of one-year follow-up diagnosis: A) Neonates that were diagnosed in the first month after delivery by microscopic blood examination (INP micromethod) (n = 19) had a median parasitemia of 1,700 Pe/mL (equivalent amounts of parasite DNA per mL); B) Infants that required a second parasitological diagnosis at six months of age (n = 10) showed a median parasitemia of around 20 Pe/mL and 500 Pe/mL at 1 and 6 months old, respectively, and C) babies with undetectable parasitemia by three blood microscopic observations but diagnosed by specific anti - *T. cruzi* serology at around 1 year old, (n = 22), exhibited a parasitemia of around 5 Pe/mL, 800 Pe/mL and 20 Pe/mL 1, 6 and 12 month after delivery, respectively. *T. cruzi* parasites were isolated by hemoculture from 19 congenitally infected children, 18 of which were genotypified as DTU TcV, (former lineage TcIId) and only one as TcI.

**Significance:**

This report is the first to quantify parasitemia levels in more than 50 children congenitally infected with *T. cruzi*, at three different diagnostic controls during one-year follow-up after delivery. Our results show that the parasite burden in some children (22 out of 51) is below the detection limit of the INP micromethod. As the current trypanocidal treatment proved to be very effective to cure *T. cruzi* - infected children, more sensitive parasitological methods should be developed to assure an early *T. cruzi* congenital diagnosis.

## Author Summary

Chagas or American Trypanosomiasis is a disease that affects around 8–10 million people in Latin America, and can be transmitted by congenital infection. In areas where the vector insect and blood transfusions are controlled, this mode of transmission is epidemiologically important, as 15,000 *Trypanosoma cruzi*-infected children are born each year. Parasitological methods are available to detect *T. cruzi* but our results suggest that more sensitive diagnostic methods should be developed, as not all newborns have high parasite burdens to be detected by microscopy. If congenital infected babies are promptly diagnosed after delivery they can be cured, as the trypanocidal treatment is very effective in children and with no significant side effects.

## Introduction

*Trypanosoma cruzi* is the causative agent of Chagas or American Trypanosomiasis, a disease that affects around 8–10 million people in Latin America [1]. In non-endemic areas, and in the absence of blood transfusion risks, the *T. cruzi* congenital transmission infection is increasing its epidemiological importance, because, according to epidemiological studies, more than 15,000 new infected babies are expected each year, and besides, the migration of *T. cruzi* infected women, mainly to North America and Europe, makes the Chagas congenital transmission a worldwide health problem [2].

The mother-to-child *T. cruzi* transmission rate in different endemic areas is variable: 10% in Paraguay [3], 3.4–8.6% in Bolivia [4]–[6], 2.3% in Chile [7], 1.4% in Brazil [8], and 7–11% in Argentina [9]–[11]. Sometimes these vertical transmission rates are underestimated, as a high percentage of children do not complete the necessary one-year follow-up to confirm *T. cruzi* infection [3], [9], [12]–[14].

The early diagnosis in infants born to seropositive women depends on the detection of blood parasites, when maternal *T. cruzi* antibodies could still be present. Ten months after delivery, the detection of specific antibodies in babies by at least two or three serological assays confirms the congenital transmission of the parasite [11], [2]. The failure to detect parasites in *T. cruzi*-infected newborns at one month of age could probably be due to a low sensitivity of the assay. This fact, in addition to the necessary one-year follow-up to diagnose this infection, claims for more sensitive screening methods to detect *T. cruzi* infected babies at birth in endemic areas.

By using a quantitative PCR, we have previously demonstrated that the parasite burden of non-pregnant women was not significantly different from that of *T. cruzi*-seropositive women pregnant with healthy children, and that parasitemia levels of women pregnant with *T. cruzi*-infected children increased about six-fold compared with that of the other two above mentioned groups of women studied [15].

In this study, we estimated the *T. cruzi* parasite burden by quantitative DNA amplification, in children diagnosed by INP micromethod at 1 and 6 months after delivery and by serology after 10 months of age. We also correlated values of parasitemia and ELISA serology in this third group of children and genotyped the isolated parasites, with the aim to further understand the congenital transmission of *T. cruzi* and improve the early diagnosis of infected newborns to allow a prompt and effective trypanocidal treatment.

## Methods

### Participants

Pregnant women and their newborns were diagnosed for *T. cruzi* infection at the Instituto Nacional de Parasitología (INP) “Dr. Mario Fatala Chaben”-ANLIS “Carlos G. Malbrán”, the reference center for diagnosis of this parasitosis in Argentina. Pregnant women were interviewed, and when confirmed as seropositive for *T. cruzi* infection, invited to diagnose their newborns after delivery. Sixty percent of women included in this study had been born in Argentina (half of them in the endemic area, in the north of the country), whereas around 35% came from Bolivia and 5% from Paraguay. All the infected pregnant women were asymptomatic and in the chronic phase of *T. cruzi* infection, resided in a non-endemic area, did not travel to the endemic area, did not receive any blood transfusions in the year before the study, and had not been previously treated with trypanocidal drugs. Seropositive pregnant women (n = 843) gave birth to 95 *T. cruzi* infected babies. The inclusion criteria for parasite burden study were that children should visit our Institution for three controls during one-year follow-up, or until they were diagnosed as *T. cruzi* infected, and only 51 children fulfilled this requirement. Most of them had GEB-blood samples available of three controls that were submitted for qPCR analysis. When 95 children were diagnosed as *T. cruzi*-infected, they were referred for trypanocidal treatment, and samples from drug-treated children were not further considered in this study.

### *T. cruzi* serological diagnosis

Mothers and their babies were serologically diagnosed at the INP 10 months after delivery, by three different tests: indirect hemagglutination, indirect immunofluorescence and enzyme-linked immunosorbent assay (ELISA), using epimastigote whole homogenate as antigen, as previously described [15]. Patients were considered *T. cruzi* infected when at least two of the serological tests were positive, according to the criteria of the World Health Organization and the approved Argentinean guidelines. Babies of 1 and 6 months of age were also tested for *T. cruzi* serology to measure specific antibodies and antibodies transferred from the mother.

### Parasitological diagnosis of *T. cruzi* infection in infants

#### Microscopic blood examination: INP micromethod

Peripheral blood from babies born to *T. cruzi*-seropositive women was obtained during three controls at 1, 6 and around 12 months of age to detect *T. cruzi* infection. The INP micromethod [16] was carried out to detect the presence of *T. cruzi* in the children's blood. Briefly, 0.5 to 1.0 mL of heparinized blood was collected by venopuncture in an Eppendorff tube, centrifuged for one minute and the buffy coat between sera and blood cells was loaded in two slides with coverslips of 22×22 mm in size, and carefully examined by microscopy at 400× for at least 30 minutes. This method proved to have 68.9% sensitive in newborns, and additionally 31.1% in infants at 6 month of age compared to xenodiagnosis [11].

#### Quantitative *T. cruzi* DNA amplification

0.5 mL of peripheral blood collected from children born to seropositive mothers were mixed with the same volume of guanidine hydrochloride 6M, EDTA 0.2 M, pH 8 (GEB) (Sigma Chemical Co., St Louis, MO, USA), kept at room temperature for 1 week and then at 4°C until use. A volume of 0.2 mL of blood-GEB was used for DNA isolation using illustra blood Mini columns (GE Healthcare Life Sciences, Uppsala, Sweden), according to the manufacturer's protocol. An internal standard of DNA extraction (2 ng) was included in each GEB sample as previously described [15]. Parasite quantification in DNA extracted samples was performed in duplicate, in an ABI 7500 thermocycler (Applied Biosystems, Carlsbad, CA, USA), amplifying a *T. cruzi* satellite sequence flanked by the Sat Fw and Sat Rv oligonucleotides [17] using the commercial kit SYBR GreenER qPCR SuperMix Universal (Invitrogen, Life Technologies, Grand Island, NY, USA). Briefly, 8 µl of DNA in 20 µl final volume reaction was subjected to DNA amplification with the following profile: 50°C for 2 min; 95°C for 10 min, and 40 cycles (95°C for 15 s, 60°C for 60 s). The *T. cruzi* CL Brener clone was used to perform a standard detection curve, spiking 10^6^ epimastigotes/mL of a seronegative blood sample - GEB mix, 10-fold diluted, and extracted as mentioned above. The Cycle threshold (Ct) of each sample after the real time PCR procedure allowed us to interpolate the parasite equivalents from a standard curve of known parasite concentrations [15]. The internal standard was also amplified with the same thermoprofile. Several points of the parasite curve, two positive samples, two negative samples and non-template DNA were included in every qPCR run. The cut off value of this method is 0,14 Pe/mL as was previously described (15), obtained after a ROC (Receiver -operating characteristic) analysis, using an available on line resource www.medcalc.org.

### Parasite hemoculture

To isolate *T. cruzi* circulating parasites, 0.5 mL of infant blood was distributed in one culture tube containing liver infusion tryptose (LIT) medium supplemented with 10% fetal calf serum and incubated at 28°C. Parasite growth was checked at 15, 30 and 45 days by microscopy and, when *T. cruzi* isolates were observed, they were immediately frozen for preservation and maintained in culture by weekly passages.

### *T. cruzi* Discrete Typing Units (DTU) PCR genotyping

DNA was extracted from hemocultured and isolated parasites with spin columns (GE Healthcare Life Sciences, Uppsala, Sweden). Parasites were PCR-genotyped targeting the intergenic regions of spliced-leader genes with primers TCC, TC1 and TC2 [18], yielding DNA amplification products of 350 bp for TcI parasites and 300 bp for TcII, TcIV and TcVI parasites. To identify other parasite DTUs another PCR assay, targeting the divergent domain of the 24S alpha rRNA gene with D71 and D72 primers was performed [19]. The reference strains Sylvio X10 (TcI), Y (TcII), M5631 (TcIII) CANIII (TcIV), Mncl2 (TcV) and CL-Brener (TcVI) were used.

### Statistical analysis

Data were presented as the medians within interquartile ranges when non-normally distributed. Differences between groups were examined by the Kruskal–Wallis test and the Dunn post-test for multiple comparisons, using GraphPad Prism 5 software (GraphPad Software, La Jolla, CA, USA). Pearson's rank correlation was used to evaluate correlations between the variables. A p value of <0.05 was considered statistically significant. The qPCR cut-off was obtained by analysis of a receiver-operating characteristic (ROC) curve, using the Med-Calc statistical software, version 11.5.0.0 as previously described [15].

### Ethics statement

This study was approved by the Ethics Committee of the ANLIS “Carlos G. Malbrán” and carried out according to the declaration of Helsinki. Informed written consent was obtained from all pregnant women included in the study before blood collection, who also have provided consent to involve their children in this study.

## Results

### Pregnant women and *T. cruzi* infected children

**Figure 1:**
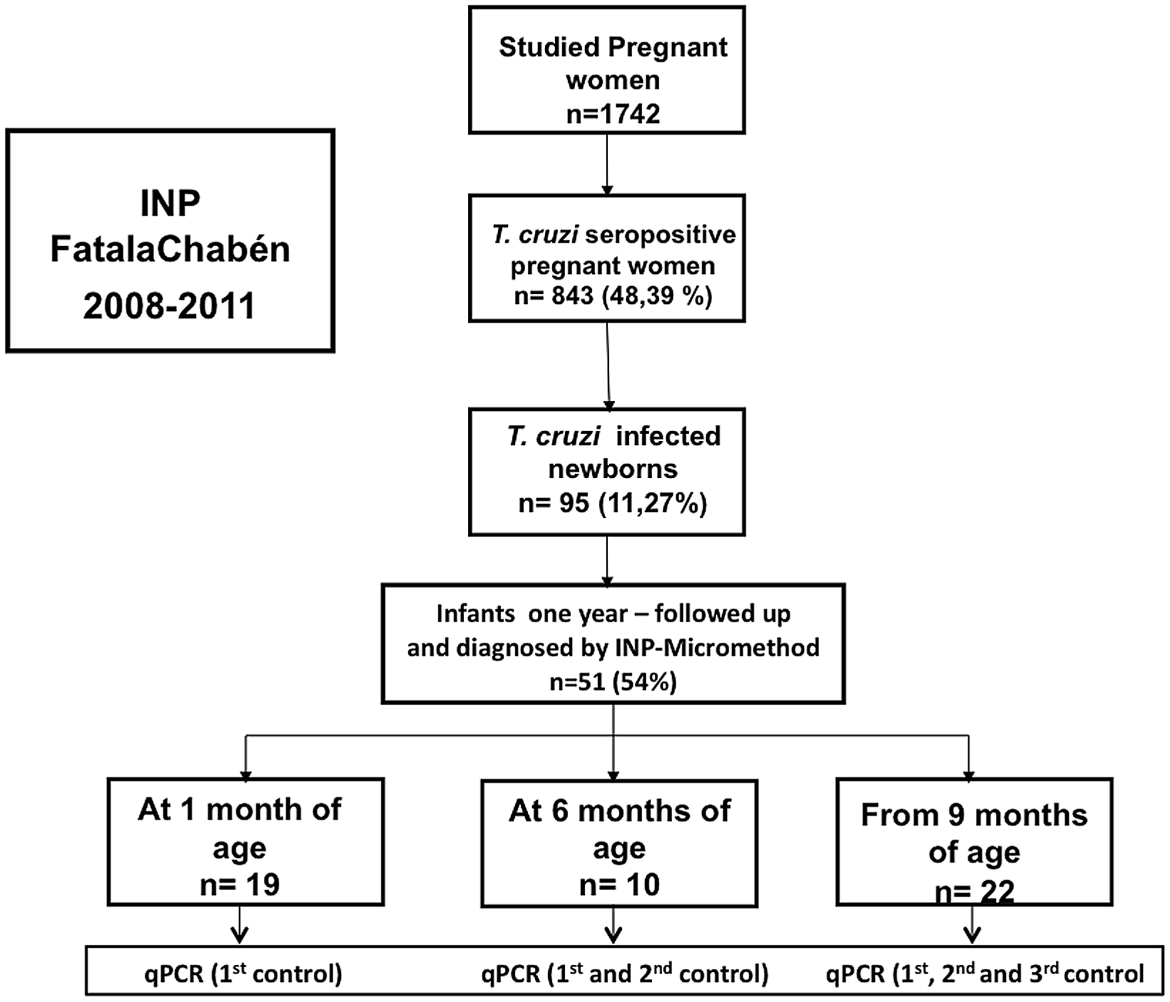
Flow diagram chart of the serological and parasitological studies in pregnant women and their infected children. *T. cruzi* quantitative DNA amplification was retrospectively performed in three groups of congenitally infected children.

A total of 1,742 pregnant women requested serology for *T. cruzi* infection at the INP “Dr. Mario Fatala Chaben”, from 2008 to 2011, 48.39% of which were diagnosed as sero-reactive. We recorded a rate of congenital parasite transmission of 11.26%, as 95 *T. cruzi*-infected infants were born to 843 seropositive mothers. Only 51 of these children were one year followed-up in our Institution for three controls (Fig. 1), at 1, 6 and around one year after delivery, according to the recommended guides for the diagnosis of congenital *T. cruzi* transmission infection, and we submitted for qPCR analysis GEB-blood samples from these 51 infected infants.

### *T. cruzi* parasite burden quantitation in three groups of infants

Infant blood samples were available at three different times of *T. cruzi* diagnosis: Group A): samples from neonates (n = 19) that were diagnosed within the first month after delivery by INP micromethod observation of bloodstream parasites Group B): samples from infants (n = 10) that required a second visit for parasitological diagnosis at six months of age and Group C): samples from babies (n = 22) diagnosed by specific anti-*T. cruzi* serology at around 1 year of life. Child blood samples were intended to be collected in every visit, so we had only one sample from each infant of Group A, who were then referred for trypanocidal treatment, we had in some cases, two samples from each child of Group B at 1 and 6 months of age, and three blood samples of each child of group C (Fig. 1 and Table 1).

**Table 1:** Quantitative parasitemia in *T. cruzi* congenitally infected children.

Children diagnosed at	A) 1 month of age	B) 6 months of age	C) 9–12 months of age
**1^st^ control by INP micromethod**	1,789 (707–5,963)		
**2^nd^ control by INP micromethod**	21 (4–255)	545 (33–2,421)	
**3^rd^ control by serology**	5 (0.2–162)	800 (141–1,318)	24 (4–805)

Parasite burden by qPCR was a retrospective work after all infected babies were referred for trypanocidal treatment. Median parasitemia (interquartile range), expressed as equivalent parasites per mL, of children diagnosed by parasitology at 1 or 6 months of age or by serology at around 12 months of age.

A quantitative PCR (qPCR) previously set up in our laboratory was performed to determine the parasitic loads in infant blood samples.

**Figure 2:**
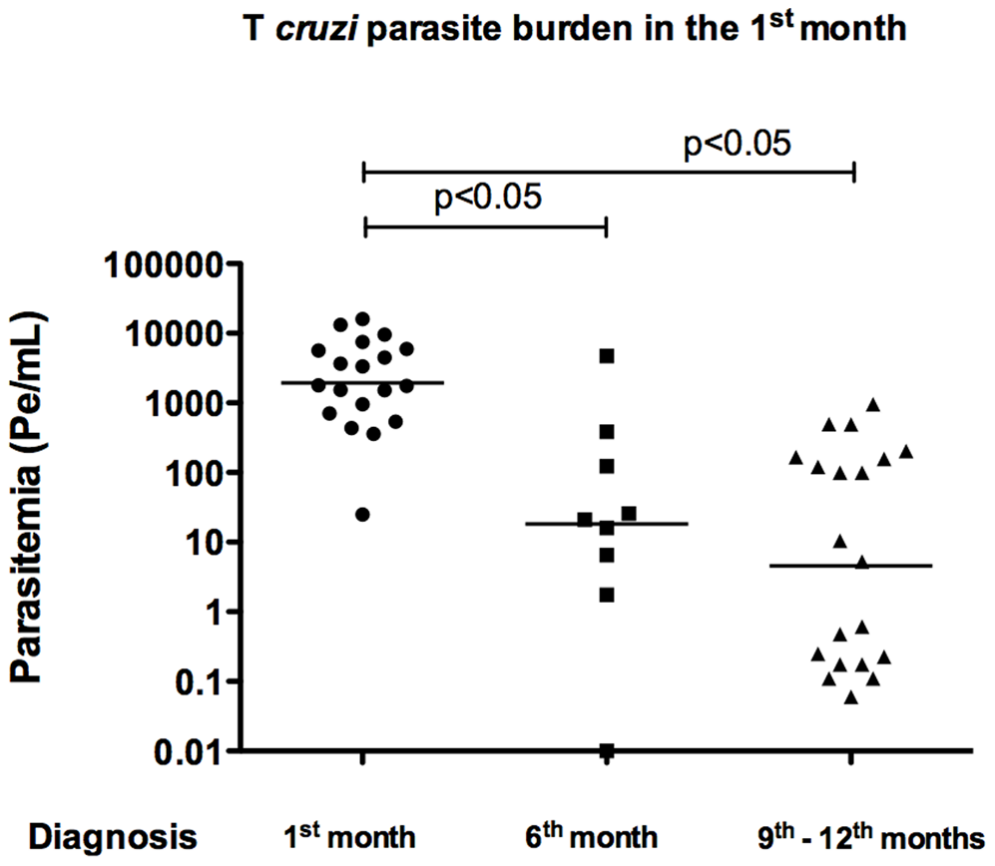
*T. cruzi* bloodstream parasite burden in newborns tested at their first month of age. In *T. cruzi* infected children diagnosed in their first, second or third control, at 1, 6 months of age and around 1 year old, repectively, parasitemia was quantified by qPCR in the sample obtained at the 1^st^ month of age. Significant differences were found in the median of the three groups assayed, by Kruskal–Wallis test using GraphPad Prism 5 software.

**Figure 3:**
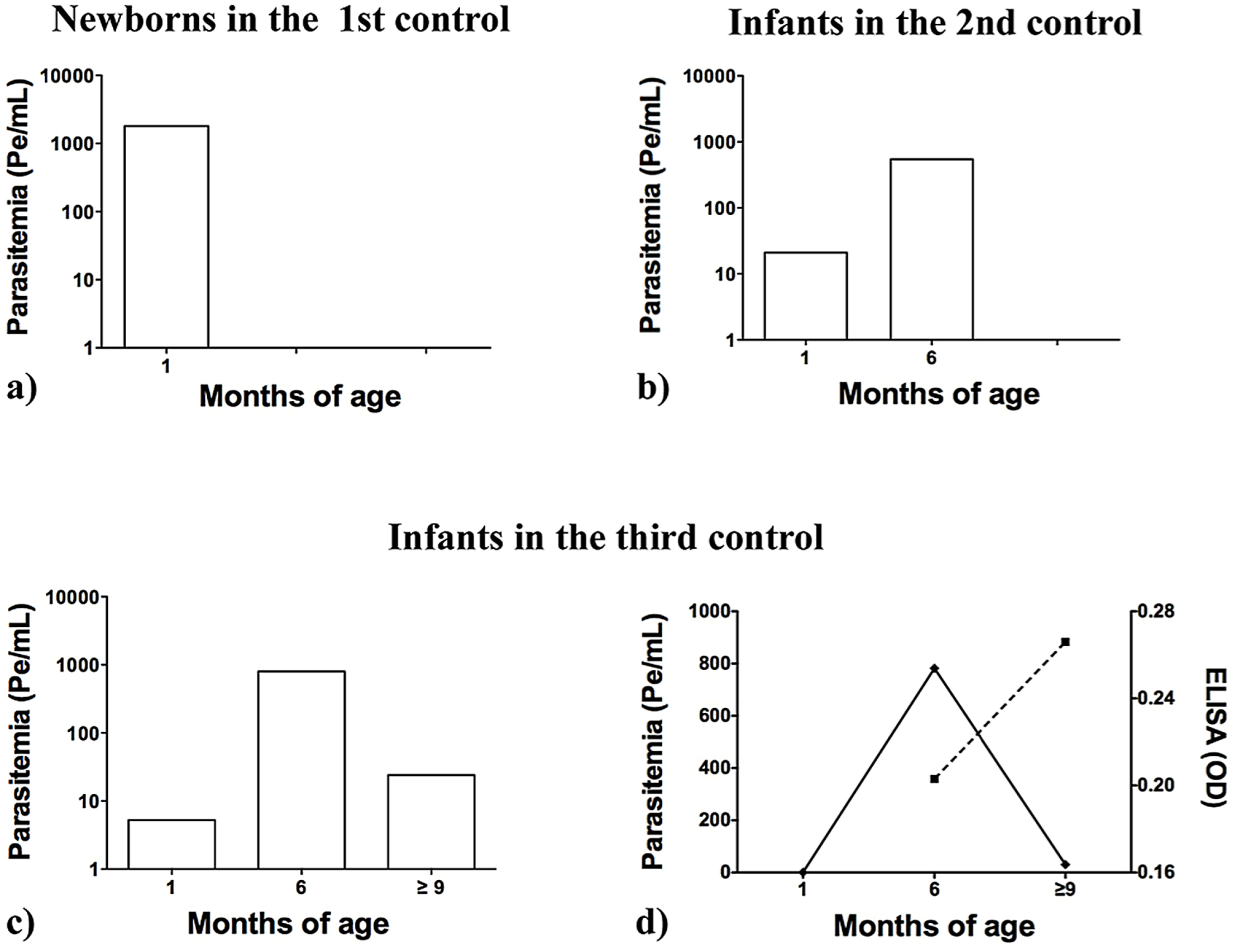
*T. cruzi* bloodstream parasite burden in infants followed-up during their first year of life. Parasitemia was quantified by qPCR in infants from group A (diagnosed at 1 month of age) (Fig. 3a); from group B (diagnosed at 6 months after delivery) (Fig. 3b); and from group C (diagnosed at around 1 year old (Fig 3c), as explained in Material and Methods. Fig. 3d shows a correlation of the parasitemia of Fig. 3c with the increment of ELISA antibodies titers (dashed line), tested at 6 and after 9 months of age.

Comparison of the bloodstream parasitic loads obtained in the three groups of children (Table 1) showed that group A neonates presented the highest levels of parasite burden 1,789 Pe/mL, (equivalent amounts of parasite DNA per mL) (Fig. 2 and 3a). Babies from group B, diagnosed as *T. cruzi*-positive at 6 months of age, showed a lower median parasitemia (21 Pe/mL) (Fig. 2 and 3b). Children from group C, with negative parasitemia by INP micromethod but diagnosed by serology at around 1 year old, exhibited the lowest parasitemia of all groups studied (5 Pe/mL) (Fig. 2 and 3c).

As shown in Table 1, Fig. 3b and Fig. 3c, children of groups B and C, who were negative for parasitological screening at 1 month of age, exhibited a higher parasitemia at 6 months of age (545 Pe/mL for group B and 800 Pe/mL for group C than that observed at 1 month of age. In group C, children serologically diagnosed at around 1 year old showed a parasite burden of 24 Pe/mL, a low parasitemia level, similar to that of these babies when they were one month old (Fig. 3c). It is worth noting that, taking into account the three controls during the one-year follow-up, the parasite burden of serologically diagnosed children of group C inversely correlated (r = 0,49) with the increment of ELISA antibodies titers, at 6 and 12 months old (Fig. 3d).

All infected infants were referred for tripanocidal treatment on the basis of parasitological diagnosis (first and second controls) or serodiagnosis (third control). Some of these treated children returned for a *T. cruzi* serology control, and when samples were available for qPCR, the parasitic burden was undetectable, below the detection limits of our quantitative method (<0.14 Pe/mL) indicating parasitological response to treatment with Benznidazole.

### *T. cruzi* DTU genotypification

Infecting *T. cruzi* parasites in 19 newborns under study were isolated from venipuncture blood samples followed by hemoculture in LIT medium. PCR genotyping showed that 18 of these isolated parasites belonged to DTU TcV (former lineage TcIId). Parasitemia could not be quantified when the sample was available at 1 month of age only in 1 out of 19 children, but we succeeded in isolating the infecting parasite by hemoculture. PCR genotyping showed that this parasite belonged to DTU TcI.

## Discussion

The aim of this study was to further understand why not every neonate born to *T. cruzi*-infected mothers can be diagnosed by microscopy observation of blood samples immediately after birth. Here, we demonstrated that only infants with very high parasitemia can be diagnosed by parasitology screening at 1 month of age, although the high efficiency exhibited by the INP micromethod, resulted in 94% sensitivity in a complete children one-year follow-up [11]. Those babies that had to return at 6 months of age for *T. cruzi* infection control were successfully diagnosed by INP micromethod observation when parasitemia increased at 6 months of age (Fig. 3b). In addition, the fact that some of the babies could only be diagnosed by serology at around 1 year old was because their parasitemia levels were under the sensitivity of the parasitological INP micromethod.

In congenitally *T. cruzi*-infected children parasitemia is usually higher than that detected in their chronic infected mothers [15], but the values of infant parasitemia is highly variable. Here, we found high parasite burdens, similar to those reported by other research groups that quantified parasitemia in congenitally infected children [20], [13] in 76 and 80% of newborns studied respectively, but certainly higher than those reported by others [21], [22], which handled lower number of samples and with low parasitemia. In some of our infant samples, showing a very high parasitemia, we confirmed values by DNA dilutions from 10 to 100 fold, to rule out dimers of satellite amplifications [17]. Moreover, our qPCR method reproduced the relationships of Cycle thresholds (Cts) and spiked blood with known parasite concentrations previously reported [17], [21].

Hemoculture is labor and time consuming and parasites must adapt to the culture medium, and it is less sensitive than molecular diagnosis methods. The fact that we successfully isolated infecting parasites in infants with very low parasitemia, expresses the uneven distribution of few parasites in a very small volume of blood sample.

Regarding parasite DTU genotyping, 18 out of 19 the parasites isolated from *T. cruzi*-infected babies were TcV, in accordance to results by a pioneer work of Virreira et al. [20] in Bolivia, and by Corrales et al. [23] in northwestern Argentina, in 34 and 18 blood samples of congenitally infected children, respectively. However, we found only one child with *T. cruzi* DTU I, who exhibited a very low parasitemia, having amplified a satellite DNA. Besides, we have previously genotyped 40 parasites isolated by hemoculture from pregnant women, mothers of *T. cruzi*-infected and non-infected children. All parasites in pregnant women were genotyped as TcV, so we found no correlation between genotype and transmission of *T. cruzi* infection, as reported by others [20], [24] when parasite genotyping was performed in areas of Northern Argentina, Bolivia and Paraguay.

New and more sensitive methods for the detection of *T. cruzi* parasites in infected newborns are needed because results of microscopic observation of blood samples are highly dependent on the operator, the time of observation and parasite motility, among others. DNA amplification of *T. cruzi* parasites is emerging as a gold standard that should hopefully be soon established as a routine method in maternity hospitals. Kinetoplast DNA seems to be a more suitable *T. cruzi* target to be amplified, because the amount of this DNA is conserved among the different DTUs. In the case of TcI parasites, the number of satellite sequences has been reported to be around 10 per cent, regarding other DTUs [17].

This parasite burden quantification in babies in three controls during a one-year follow-up explains the difficulties to succeed in diagnosing the parasite immediately after birth and stresses the need to increase the sensitivity of new parasitological diagnosis methods, for the prompt and effective trypanocidal treatment of congenitally infected children.

## Supporting Information

Checklist S1 STROBE checklist.
(DOCX)
